# Risk factors for chronic pouchitis in patients with ulcerative colitis following ileal pouch-anal anastomosis: a meta-analysis

**DOI:** 10.3389/fmed.2026.1886122

**Published:** 2026-08-17

**Authors:** Zhen Meng, Hongli Yi, Shuxia Yu, Jing Chen, Minhui Sun

**Affiliations:** Shandong Provincial Hospital Affiliated to Shandong First Medical University, Jinan, Shandong, China

**Keywords:** chronic pouchitis, ileal pouch-anal anastomosis, meta-analysis, risk factors, systematic review, ulcerative colitis

## Abstract

**Background:**

Chronic pouchitis is one of the common complications following ileal pouch-anal anastomosis (IPAA) in patients with ulcerative colitis (UC), and its pathogenesis and risk factors remain incompletely understood. Therefore, this study aims to evaluate the association between multiple potential risk factors and the occurrence of postoperative chronic pouchitis in UC patients through a systematic meta-analysis.

**Methods:**

Databases including PubMed, Embase, Cochrane Library, and Web of Science were searched up to January 20, 2026. Eligible cohort studies and case-control studies were included. Study quality was assessed using the Newcastle–Ottawa Scale (NOS). Meta-analysis employed a random-effects model to calculate the odds ratio (OR) and 95% confidence interval (CI) for each factor associated with chronic pouchitis. All statistical analyses were performed using Stata 15.0 (StataCorp LLC, College Station, TX, USA) software.

**Results:**

Thirteen studies involving 4,264 patients with UC undergoing IPAA were included, of whom 916 developed chronic pouchitis. The pooled analysis showed that female sex (OR = 1.36, 95% CI: 1.05–1.76), current smoking (current smokers vs. never-smokers; OR = 2.46, 95% CI: 1.28–4.75), primary sclerosing cholangitis (OR = 2.30, 95% CI: 1.43–3.72), preoperative systemic steroid use (OR = 1.61, 95% CI: 1.10–2.36), immunosuppressive therapy (OR = 1.32, 95% CI: 1.02–1.70), and extraintestinal manifestations (OR = 3.09, 95% CI: 1.65–5.77) were associated with increased risk of chronic pouchitis. Increasing age was associated with chronic pouchitis in the primary analysis; however, this association was not statistically significant after trim-and-fill adjustment, suggesting potential influence of publication bias.

**Conclusions:**

Several clinical factors, including female sex, current smoking, primary sclerosing cholangitis, preoperative steroid or immunosuppressive use, and extraintestinal manifestations, are associated with an increased risk of chronic pouchitis after IPAA. The association between age and chronic pouchitis appears unstable and should be interpreted cautiously. These findings may assist in identifying high-risk patients and guiding postoperative surveillance; however, further prospective studies are needed to confirm these associations.

## Background

Ulcerative colitis (UC) is a chronic, recurrent inflammatory bowel disease primarily affecting the colon and rectum (1). For some patients with severe or refractory UC, when traditional drug therapies fail to effectively control the condition, surgical intervention becomes a common treatment option (2). Ileal pouch-anal anastomosis (IPAA), the standard surgical treatment for UC, involves removing the diseased colon and rectum and reconstructing an “ileal pouch” to replace them (3). This procedure significantly improves patients' quality of life and bowel function. However, while most patients achieve symptom relief after IPAA, approximately 40%−60% experience postoperative complications (4, 5). Among these, chronic pouchitis stands as one of the most common and challenging complications.

Chronic pouchitis refers to persistent inflammation of the ileal pouch, presenting with symptoms such as diarrhea, abdominal pain, mucus secretion, and even bloody stools (6). Severe cases may significantly impair quality of life, necessitate reoperation, or lead to more serious complications (7). The occurrence of chronic pouchitis not only affects long-term patient prognosis but may also result in multiple hospitalizations, increasing the burden on healthcare systems (8). Therefore, identifying and predicting risk factors for chronic pouchitis is crucial for guiding clinicians in risk assessment and personalized management (9).

Current research indicates multiple factors may be associated with chronic pouchitis development. Patient age, gender, smoking history, and preoperative disease activity are all considered potential risk factors (10). Notably, smoking history is recognized as a significant influence on postoperative chronic pouchitis in UC patients, with studies showing a markedly increased risk among smokers. Additionally, factors such as surgical technique selection, ileal pouch structure, postoperative immunosuppressive therapy, and antibiotic use are also closely associated with chronic pouchitis development. Studies indicate that postoperative infection or inflammatory responses in the ileal pouch may exacerbate pouchitis severity (11, 12). Among patients receiving postoperative immunosuppressive therapy, long-term use of immunosuppressive agents may be linked to chronic pouchitis progression.

However, despite extensive research on chronic pouchitis risk factors, existing findings remain controversial and inconsistent (13). Differences in study design, sample size, assessment criteria, and patient populations lead to substantial variability in risk factor interpretation. Consequently, factors identified as high-risk in individual studies may fail to demonstrate significant associations in comprehensive analyses.

To comprehensively evaluate and identify the primary risk factors for chronic pouchitis, this study aims to synthesize and analyze relevant literature, thereby drawing conclusions from a broader sample and more rigorous analysis. By examining patients' clinical characteristics and various preoperative and postoperative factors, this study identifies independent risk factors closely associated with chronic pouchitis. This provides a theoretical basis for early clinical identification and management of high-risk patients. It will assist clinicians in implementing early intervention and management for high-risk individuals, thereby reducing the incidence of chronic pouchitis, improving patient prognosis, and offering additional guidance for the surgical treatment of UC.

## Methods

This systematic review and meta-analysis have been registered with PROSPERO under registration number CRD420251247351, dated December 5, 2025. All research methodologies and data analysis plans were executed strictly according to the content submitted during registration, ensuring transparency and reproducibility of the study. Through registration with PROSPERO, we guaranteed the transparency and standardization of research procedures, with each step traceable and verifiable. This registration complies with the PRISMA guidelines (14) to ensure high-quality implementation of this systematic review and meta-analysis.

### Literature search strategy

To ensure breadth and comprehensiveness, we conducted literature searches across multiple academic databases, including PubMed, Embase, the Cochrane Library, and Web of Science. The search timeframe spanned from each database's inception to January 20, 2026. Search terms included: “ulcerative colitis,” “chronic pouchitis,” and “risk factors.” Additionally, we supplemented the search by manually reviewing reference lists of relevant publications. The specific search strategy is detailed in Supplementary Table S1.

### Inclusion and exclusion criteria

Inclusion criteria include: (1) Study subjects are patients with UC undergoing ileal pouch-anal anastomosis; (2) Study design is case-control or cohort studies; (3) Studies providing relevant risk factors for chronic pouchitis; (4) Studies presenting statistical data on risk factors, such as odds ratios (OR) and 95% confidence intervals (CI). Exclusion criteria include: (1) Studies involving patients without UC; (2) Studies with insufficient data or unable to extract valid information; (3) Animal studies, case reports, or conference abstracts.

### Data extraction

During data extraction, two independent researchers followed predefined criteria to ensure accuracy and consistency of information. In cases of disagreement between researchers, a third-party expert reached a consensus through discussion. Extracted data included study details (authors, publication year, study design, location), patient clinical characteristics (age, gender, number of chronic pouchitis cases), surgical type, and regression models. All extracted data underwent rigorous verification to ensure no omissions or errors. When multiple estimates were reported, we preferentially extracted adjusted odds ratios (ORs) with 95% confidence intervals (CI) from multivariable regression models. If adjusted estimates were unavailable, the corresponding unadjusted ORs were used. Only one effect estimate per study was included in the pooled analysis to ensure consistency. The indication for colectomy was variably reported across the included studies. Some studies distinguished medically refractory disease from dysplasia or colorectal cancer, whereas others combined refractory disease with acute severe or fulminant colitis or used broader categories. Because these categories were not sufficiently comparable across studies, colectomy indication was summarized descriptively rather than pooled as an additional risk factor. The available study-level information is presented in Supplementary Table S3.

### Quality assessment

In this study, we employed the Newcastle-Ottawa Scale (NOS) (15) to assess the quality of included studies. The NOS primarily encompasses three domains: selection, comparison, and outcome. In the selection domain, we evaluated the representativeness of study subjects and the appropriateness of control groups to ensure comparability between cases and controls. The comparison domain assessed whether studies effectively controlled for potential confounding factors such as age, gender, and smoking history. The outcomes section focused on the consistency of outcome definitions and measurement methods for chronic pouchitis, ensuring accuracy and reproducibility of results. Each section was scored on a 0–1 scale, yielding a final score ranging from 0 to 9, with higher scores indicating better study quality. Low-quality studies were excluded via sensitivity analysis to minimize their impact on conclusions. This quality assessment ensured the reliability of included studies and enhanced the scientific rigor and accuracy of the findings. The certainty of evidence for each outcome was assessed using the Grading of Recommendations Assessment, Development and Evaluation (GRADE) approach, considering study limitations, inconsistency, indirectness, imprecision, and publication bias.

### Statistical analysis

All statistical analyses were performed using Stata 15.0 software. For risk factor analysis, we extracted adjusted OR and their 95% CI as effect measures from each study and uniformly converted them to log values for pooled calculations. Given potential clinical and methodological differences among included studies (study design, patient characteristics, and diagnostic criteria), a random-effects model was employed for all meta-analyses. Model parameters utilized the DerSimonian–Laird method to calculate between-study variance (τ^2^) for more robust and conservative effect estimates.

Inter-study heterogeneity was assessed using Cochran's *Q* test and the I^2^ statistic. An *I*^2^ value below 25% indicates low heterogeneity, 25%−50% indicates moderate heterogeneity, and above 50% indicates high heterogeneity. When significant heterogeneity was present, we validated findings through sensitivity analyses by sequentially excluding each study (i.e., leave-one-out method) to assess robustness. Additionally, we ensured conclusion reliability by testing consistency of results across different effect model parameter estimation methods.

Bias assessment was performed visually via funnel plots and statistically validated using Egger regression and Begg's rank correlation tests. When potential bias was identified, further correction was applied using the “trim-and-fill method.” All statistical tests were two-sided, with results considered statistically significant at *P* < 0.05.

## Results

### Study selection

**Figure 1:**
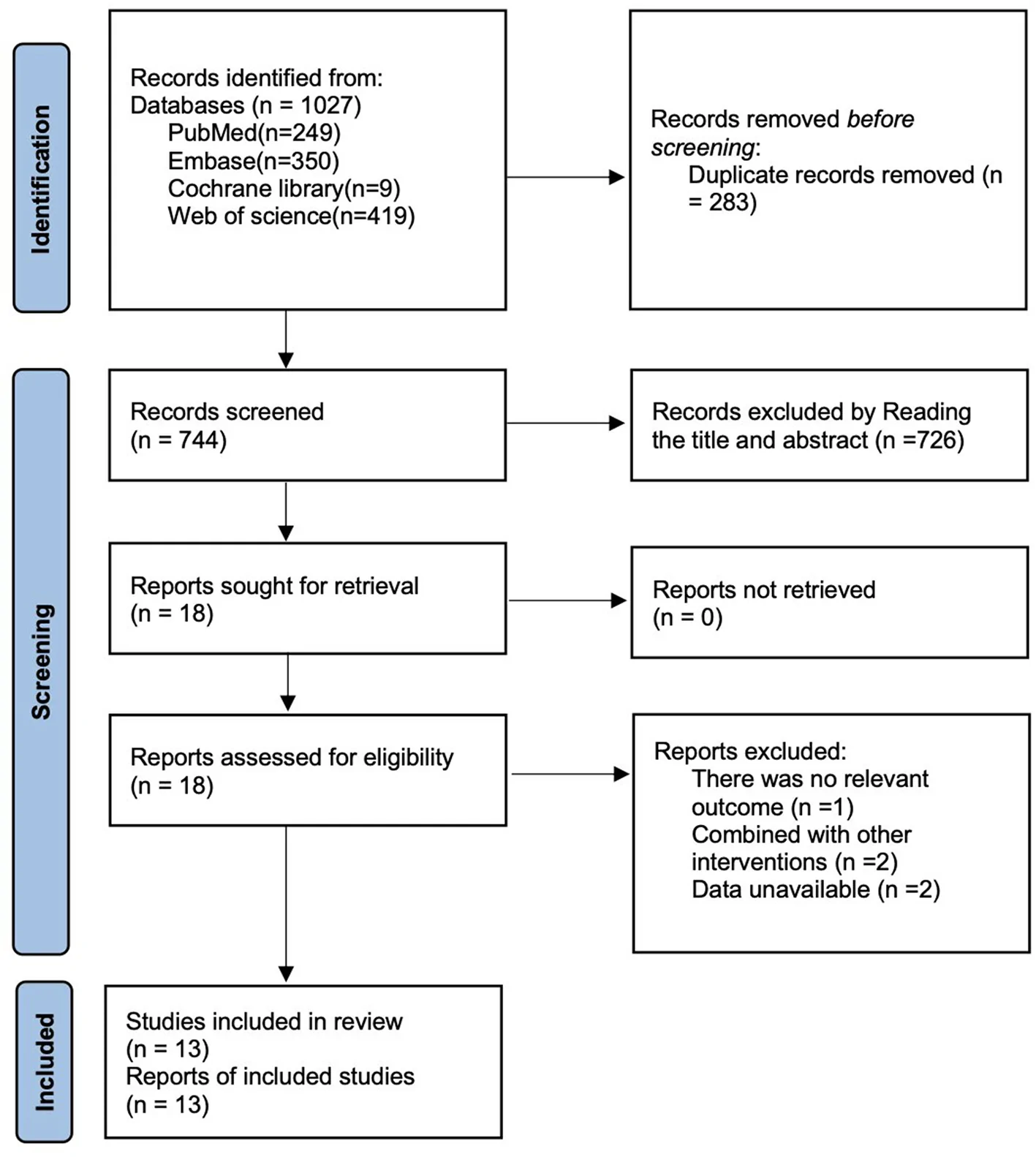
PRISMA flow diagram of study selection.

The initial literature search identified 1,027 potentially relevant records across four databases (PubMed, Embase, Cochrane Library, and Web of Science). After eliminating 283 duplicate publications, 744 unique articles remained for title and abstract screening, the full texts of 18 potentially eligible articles were subsequently retrieved and carefully reviewed, following the full-text assessment, five studies were excluded. Finally, 13 studies (16–28) were included in the systematic review and meta-analysis. The detailed selection procedure is illustrated in Figure 1. The indication for colectomy was variably reported across the included studies. Some studies distinguished medically refractory disease from dysplasia or colorectal cancer, whereas others combined refractory disease with acute severe or fulminant colitis or used broader categories. Because these categories were not sufficiently comparable across studies, colectomy indication was summarized descriptively rather than pooled as an additional risk factor. The available study-level information is presented in Supplementary Table S3.

### Characteristics of the included studies

A total of 13 studies published between 2003 and 2025 were included in this meta-analysis, comprising 4,264 patients with UC who underwent IPAA, among whom 916 developed chronic pouchitis. The studies were conducted in the United States, Japan, the United Kingdom, Korea, Belgium, and Israel, and included 12 cohort studies and one case-control study. Sample sizes ranged from 58 to 1,136, with mean patient ages between 29 and 51.2 years. Logistic regression was the most used statistical method, while one study applied Cox univariate analysis. Among studies reporting follow-up, the follow-up period ranged from a 2-year observation window to a median of 125 months; two studies did not report follow-up duration. Detailed study characteristics, including study-specific follow-up periods, are shown in Table 1.

**Table 1:** Characteristics of the studies included in the meta-analysis.

References	Study design	Country	Sample size	Number of chronic pouchitis	Gender (M/F)	Mean age (years)	Type of surgery	Regression	Follow-up period
Abdelrazeq et al. (16)	Cohort study	UK	198	29	116/82	31.4	IPAA	Logistic regression	Mean 64 months (range 12–180)
Akiyama et al. (17)	Cohort study	Japan	392	125	224/168	32	IPAA	Logistic regression	Median 8.5 years (IQR 4.8–12.7)
Baek et al. (18)	Cohort study	Korea	232	74	133/99	37	IPAA	Cox univariate analysis	NR for total cohort; CP subgroup: median 99 months (IQR 44.8–164.5)
Barnes et al. (19)	Cohort study	USA	594	111	326/268	41.3	IPAA	Logistic regression	Up to 2 years
Bertucci Zoccali et al. (20)	Case-control	USA	411	166	240/171	36	IPAA	Logistic regression	Median 62 months
Ferrante et al. (21)	Cohort study	Belgium	172	80	105/67	39.1	IPAA	Logistic regression	Median 6.7 years (IQR 3.7–10.5)
Fleshner et al. (22)	Cohort study	USA	186	23	112/74	38	IPAA	Logistic regression	Median 24 months (range 3–117)
Gorrepati et al. (23)	Cohort study	USA	353	126	211/141	51.2	IPAA	Logistic regression	NR
Hashavia et al. (24)	Cohort study	Israel	201	63	106/95	35	IPAA	Logistic regression	Mean 107 months
Hata et al. (26)	Cohort study	Japan	58	9	30/28	32.1	IPAA	Logistic regression	NR
Hata et al. (25)	Cohort study	Japan	100	12	60/40	29.33	IPAA	Logistic regression	Median 125 months (range 5–251)
Kasashima et al. (27)	Cohort study	Japan	1,136	67	674/462	42	IPAA	Logistic regression	Median 77.6 months
Okita et al. (28)	Cohort study	Japan	231	31	128/103	29	IPAA	Logistic regression	Median 1,882.5 days (range 31–4,465)

CP, chronic pouchitis; IPAA, ileal pouch–anal anastomosis; IQR, interquartile range; NR, not reported.

### Quality assessment

The methodological quality of the included studies was evaluated using the NOS. The NOS scores of the included studies ranged from 7 to 9, indicating moderate to high methodological quality. Most studies achieved high scores in the selection and outcome/exposure assessment domains. Several studies received slightly lower scores in the comparability domain due to limited adjustment for confounding factors. Detailed NOS assessment results are presented in Table 2. The certainty of evidence was assessed using the GRADE approach (Supplementary Table S2) and ranged from very low to moderate. Primary sclerosing cholangitis and extraintestinal manifestations were rated as moderate certainty. Female sex, smoking, preoperative systemic steroid use, and immunosuppressive therapy were rated as low certainty due to study design limitations, heterogeneity, and residual confounding. The association between age and chronic pouchitis was graded as very low certainty because of publication bias and instability after trim-and-fill adjustment.

**Table 2:** Quality assessment of the included studies using the Newcastle–Ottawa Scale (NOS).

References	Representativeness of the exposed group	Selection of non-exposed groups	Determination of exposure factors	Identification of outcome indicators not yet to be observed at study entry	Comparability of exposed and unexposed groups considered in design and statistical analysis	Design and statistical analysis	Adequacy of the study's evaluation of the outcome	Adequacy of follow-up in exposed and unexposed groups	Total scores
Cohort study									
Abdelrazeq et al. (16)	^*^	^*^	^*^	^*^	^**^	^*^	^*^	^*^	9
Akiyama et al. (17)	^*^	^*^	^*^	/	^**^	^*^	^*^	^*^	8
Baek et al. (18)	^*^	^*^	^*^	/	^**^	^*^	^*^	^*^	8
Barnes et al. (19)	^*^	^*^	^*^	^*^	^**^	^*^	^*^	^*^	9
Ferrante et al. (21)	^*^	^*^	^*^	^*^	^**^	^*^	^*^	^*^	9
Fleshner et al. (22)	^*^	^*^	^*^	/	^*^	^*^	^*^	^*^	7
Gorrepati et al. (23)	^*^	^*^	^*^	/	^**^	^*^	^*^	^*^	8
Hashavia et al. (24)	^*^	^*^	^*^	/	^*^	^*^	^*^	^*^	7
Hata et al. (26)	^*^	^*^	^*^	/	^**^	^*^	^*^	^*^	8
Hata et al. (25)	^*^	^*^	^*^	/	^*^	^*^	^*^	^*^	7
Kasashima et al. (27)	^*^	^*^	^*^	^*^	^**^	^*^	^*^	^*^	9
Okita et al. (28)	^*^	^*^	^*^	/	^*^	^*^	^*^	^*^	7
**References**	**Is the case definition adequate?**	**Representativeness of the cases**	**Determination of control group**	**Definition of controls**	**Comparability of cases and controls based on the design or analysis**	**Ascertainment of exposure**	**Same method of ascertainment for cases and controls**	**Non response**	**Total scores**
Case control									
Bertucci Zoccali et al. (20)	^*^	^*^	^*^	^*^	^**^	^*^	^*^	^*^	9

^*^ one score, ^**^ two score.

### Meta-analysis results

#### Increasing age

**Figure 2:**
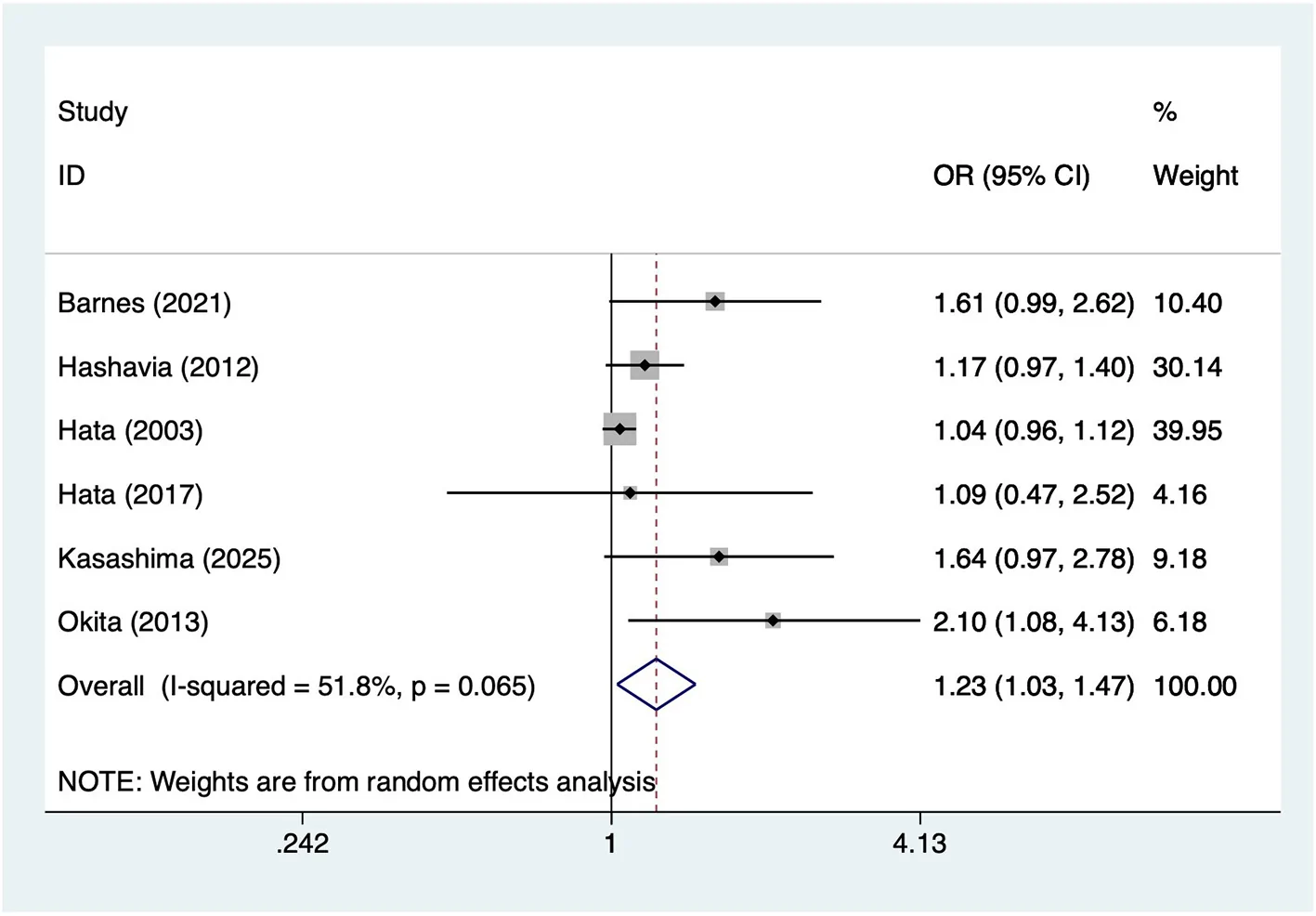
Forest plot of age and chronic pouchitis risk.

Six studies evaluated the association between age and the risk of chronic pouchitis. Moderate heterogeneity was observed among the studies (*I*^2^ = 51.8%, *p* = 0.065). The pooled analysis (Figure 2) indicated that increasing age was associated with an increased risk of chronic pouchitis (OR = 1.23, 95% CI: 1.03–1.47). Sensitivity analysis (Supplementary Figure S1) performed by sequential exclusion of individual studies suggested that the overall direction of the associations remained stable.

#### Female

**Figure 3:**
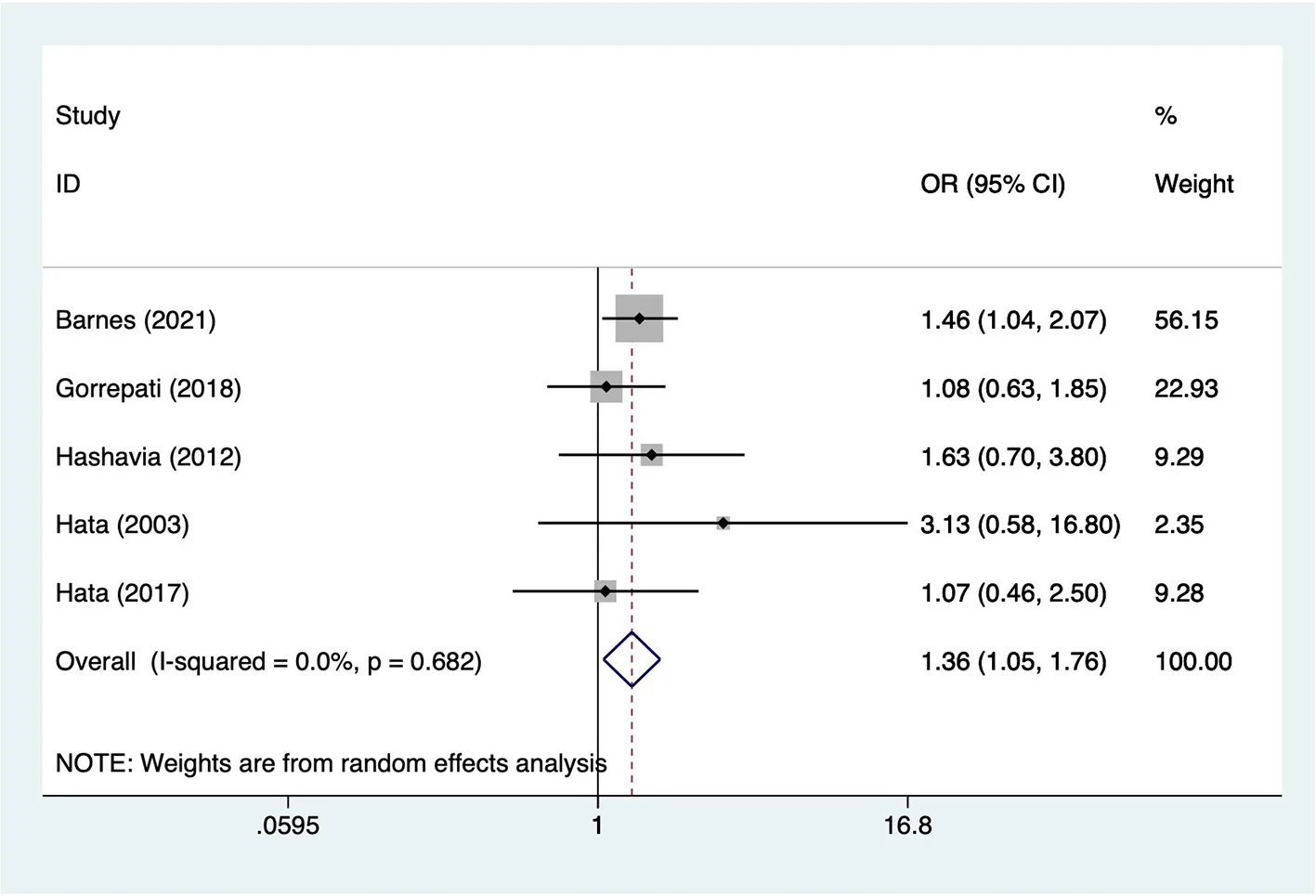
Forest plot of female sex and chronic pouchitis risk.

Five studies evaluated the association between female and the risk of chronic pouchitis. Moderate heterogeneity was observed among the studies (*I*^2^ = 0%, *p* = 0.682). The pooled analysis (Figure 3) indicated that female was associated with an increased risk of chronic pouchitis (OR = 1.36, 95% CI: 1.05–1.76).

#### Current smoking

**Figure 4:**
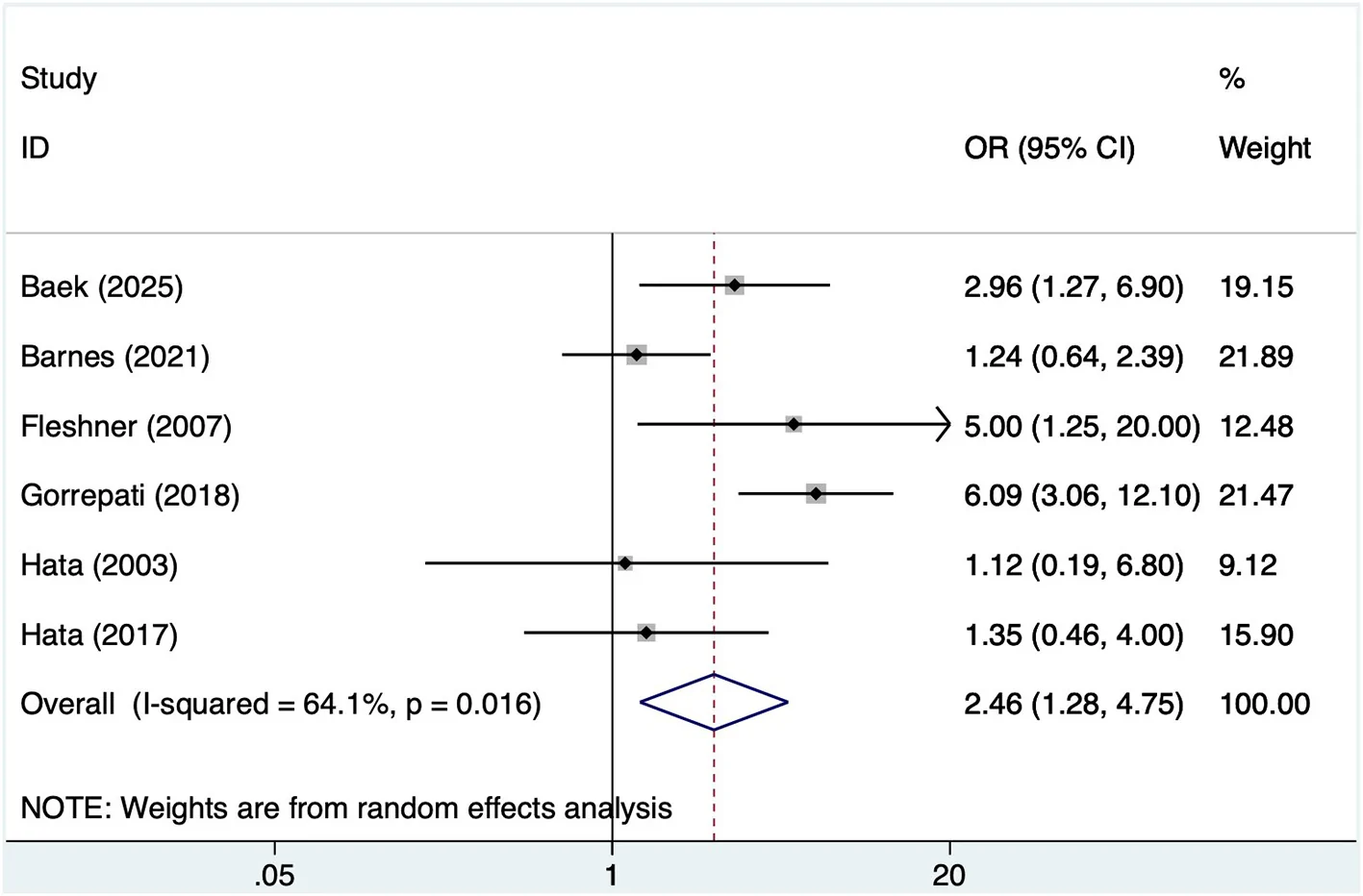
Forest plot of smoking and chronic pouchitis risk.

Six studies compared current smokers with never-smokers. No heterogeneity was observed among the studies (*I*^2^ = 0%, *p* = 0.682). The pooled analysis (Figure 4) indicated that current smoking was associated with an increased risk of chronic pouchitis (OR = 2.46, 95% CI: 1.28–4.75). Sensitivity analysis results (Supplementary Figure S2) indicate that the pooled effect size did not change significantly after excluding any single study, demonstrating the robustness of the overall conclusion.

#### Primary sclerosing cholangitis

**Figure 5:**
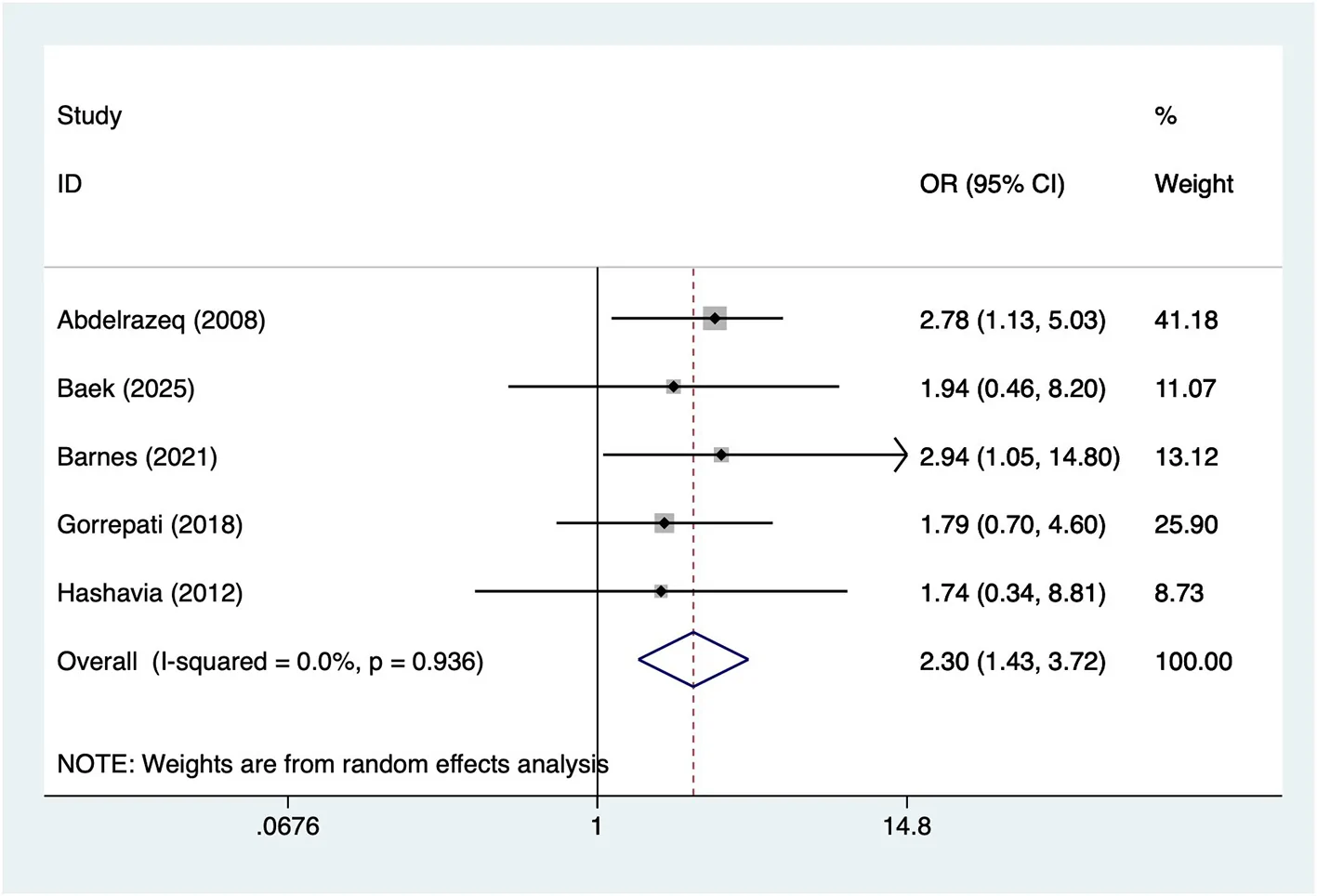
Forest plot of primary sclerosing cholangitis and chronic pouchitis risk.

Five studies evaluated the association between primary sclerosing cholangitis and the risk of chronic pouchitis. Moderate heterogeneity was observed among the studies (*I*^2^ = 0%, *p* = 0.936). The pooled analysis (Figure 5) indicated that primary sclerosing cholangitis was associated with an increased risk of chronic pouchitis (OR = 2.30, 95% CI: 1.43–3.72).

#### Preoperative systemic steroids

**Figure 6:**
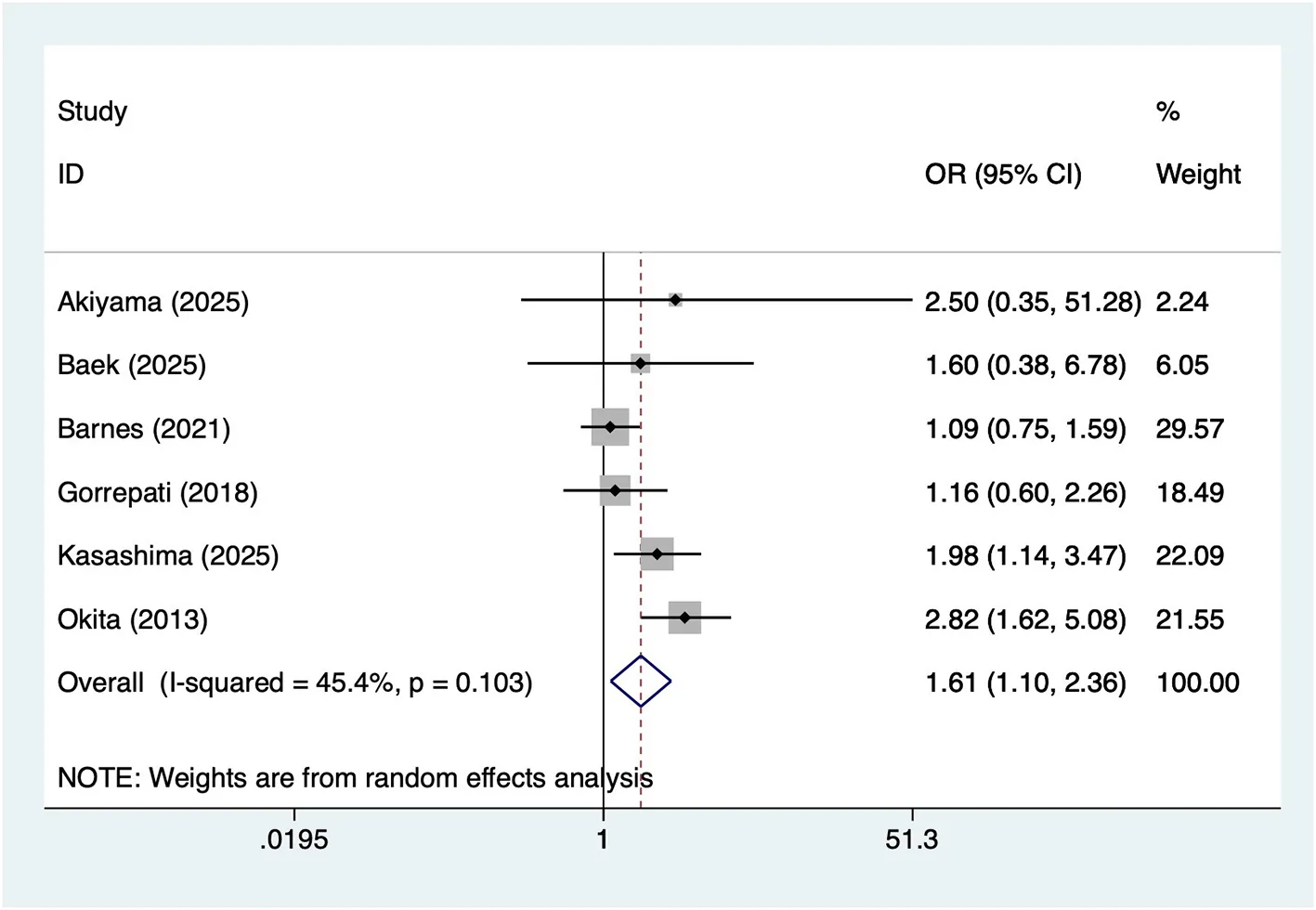
Forest plot of preoperative systemic steroid use and chronic pouchitis risk.

Six studies evaluated the association between preoperative systemic steroids and the risk of chronic pouchitis. Moderate heterogeneity was observed among the studies (*I*^2^ = 45.4%, *p* = 0.103). The pooled analysis (Figure 6) indicated that preoperative systemic steroids was associated with an increased risk of chronic pouchitis (OR = 1.61, 95% CI: 1.10–2.36).

#### Preoperative immunosuppressive therapy

**Figure 7:**
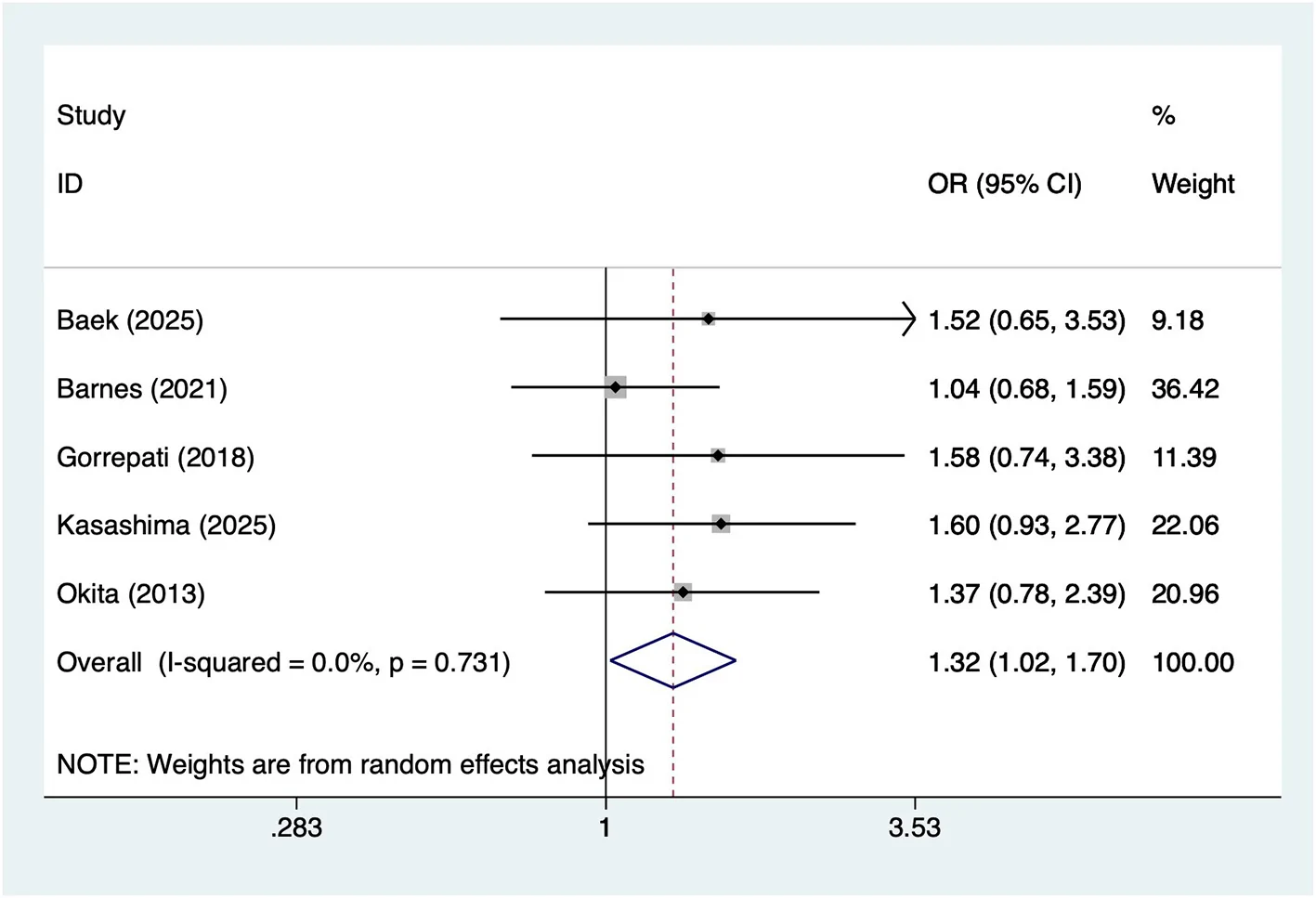
Forest plot of preoperative immunosuppressive therapy and chronic pouchitis risk.

Five studies evaluated the association between preoperative immunosuppressive therapy and the risk of chronic pouchitis. Moderate heterogeneity was observed among the studies (*I*^2^ = 0%, *p* = 0.731). The pooled analysis (Figure 7) indicated that preoperative systemic steroids was associated with an increased risk of chronic pouchitis (OR = 1.32, 95% CI: 1.02–1.70).

#### Extraintestinal manifestations

**Figure 8:**
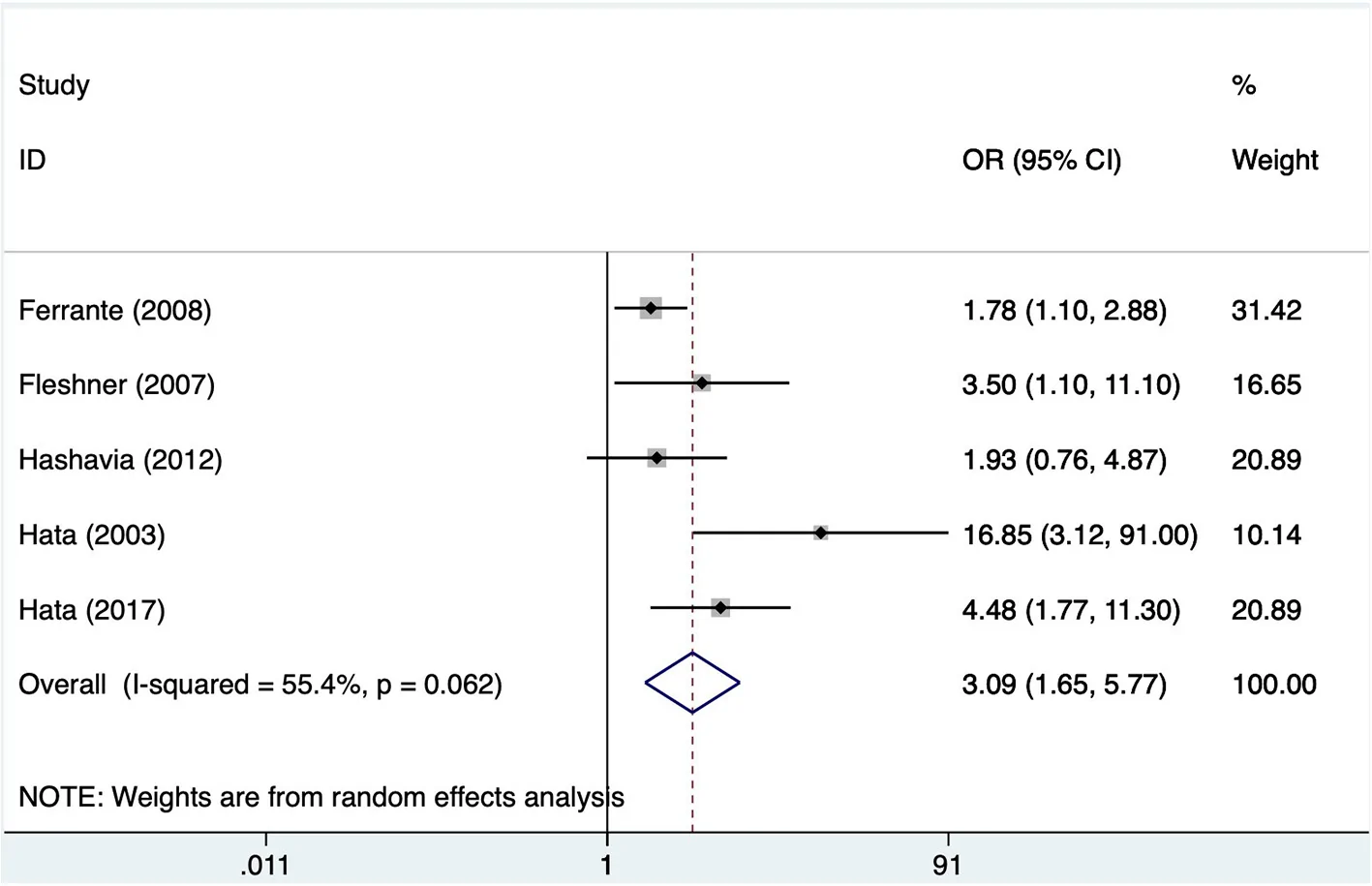
Forest plot of extraintestinal manifestations and chronic pouchitis risk.

Five studies evaluated the association between EIMs and the risk of chronic pouchitis. The definition and composition of EIMs varied among the studies. Where specified, the composite EIM variable included musculoskeletal, dermatologic, ocular, and hepatobiliary manifestations. Primary sclerosing cholangitis was additionally evaluated as a separate exposure when sufficient data were available. Moderate heterogeneity was observed among the studies (*I*^2^ = 55.4%, *p* = 0.062). The pooled analysis (Figure 8) indicated that the presence of any reported EIM was associated with an increased risk of chronic pouchitis (OR = 3.09, 95% CI: 1.65–5.77). Sensitivity analysis results (Supplementary Figure S3) showed that the pooled effect estimate did not change substantially after excluding any single study. Because most studies did not report separate effect estimates for individual EIM subtypes, this pooled result should be interpreted as the association with a composite EIM variable rather than with any specific manifestation.

#### Publication bias

Publication bias was evaluated using funnel plots and Egger's test. The results (Supplementary Figures S4–S10) suggested potential publication bias for increasing age (Egger's test, *P* = 0.025), given the limited number of studies (*n* = 6), the Egger's test may have low statistical power, and the observed asymmetry should be interpreted with caution. No significant publication bias was observed for smoking (*P* = 0.836), primary sclerosing cholangitis (*P* = 0.525), preoperative systemic steroid use (*P* = 0.476), preoperative immunosuppressive drug use (*P* = 0.151), or extraintestinal manifestations (*P* = 0.060). The trim-and-fill method identified two potentially missing studies. After adjustment (Supplementary Figures S11), the pooled estimate decreased and lost statistical significance (adjusted estimate = 0.132, 95% CI: −0.053–0.316, *P* = 0.163), suggesting that publication bias may have influenced the original results.

## Discussion

### Key finding

Chronic pouchitis represents one of the most frequent long-term complications following IPAA in patients with UC. Although this surgical procedure can provide satisfactory disease control for many patients, a proportion continue to experience persistent pouch inflammation after surgery. The factors that predispose individuals to chronic pouchitis remain incompletely understood. By synthesizing data from 13 studies involving more than four thousand patients, the present analysis identified several variables that appear to be associated with an increased likelihood of chronic pouchitis, including female sex, smoking, primary sclerosing cholangitis, preoperative exposure to systemic steroids or immunosuppressive agents, and the presence of extraintestinal manifestations. Age also showed an association in the primary analysis, although this relationship became less evident after adjustment for potential publication bias.

The relatively strong association observed between primary sclerosing cholangitis and chronic pouchitis is consistent with previous clinical observations suggesting that patients with UC and concomitant primary sclerosing cholangitis may represent a distinct disease phenotype (29, 30). A 2025 meta-analysis by Khoo et al. (31), which included 11 publications, similarly identified primary sclerosing cholangitis and extraintestinal manifestations as risk factors for chronic pouchitis, but did not find significant associations for sex, age, or immunomodulator use. Several methodological differences may account for these divergent results. Khoo et al. searched through October 2023, whereas the present review searched through January 2026. Our later search captured three eligible cohorts published in 2025 and, together with differences in eligibility decisions, yielded 13 studies overall vs. 11. Differences in chronic pouchitis definitions, exposure coding, and the availability or selection of adjusted vs. unadjusted effect estimates may also shift pooled results; this is consequential because only five to six studies contributed to each of our pooled estimates. Furthermore, the associations for female sex and immunosuppressive therapy in the present analysis were modest (ORs 1.36 and 1.32, respectively) and may be sensitive to study selection, residual confounding, and covariate adjustment. The non-significant age estimate reported by Khoo et al. is also consistent with the instability of our age finding after trim-and-fill adjustment. Taken together, the concordant findings for primary sclerosing cholangitis and extraintestinal manifestations appear more robust, whereas the associations with female sex, age, and immunosuppressive therapy require confirmation in larger prospective cohorts. Several mechanisms have been proposed to explain this relationship. Alterations in bile acid metabolism, immune signaling pathways, and intestinal microbiota composition have all been implicated in linking hepatobiliary disease with intestinal inflammation (32). In addition, PSC-associated UC has been reported to demonstrate distinct immunologic characteristics, including increased mucosal immune activation and altered microbial diversity (33). These factors may contribute to a persistent pro-inflammatory environment within the ileal pouch even after removal of the colon, thereby increasing susceptibility to chronic pouch inflammation (34).

Extraintestinal manifestations were also associated with a higher risk of chronic pouchitis. In inflammatory bowel disease, extraintestinal manifestations are often considered indicators of systemic immune dysregulation rather than isolated intestinal disease (35). Their presence may therefore reflect a broader inflammatory phenotype that extends beyond the colon. Previous studies (36, 37) have suggested that patients with extraintestinal manifestations tend to exhibit heightened immune activity and a greater burden of inflammatory complications. This systemic component may persist even after colectomy, which could partly explain why these patients appear more prone to developing chronic pouch inflammation.

An association was also observed between preoperative medical therapy and the occurrence of chronic pouchitis. Patients who require systemic corticosteroids or immunosuppressive agents prior to surgery often have more severe or treatment-refractory disease. Consequently, these medications may serve primarily as indicators of underlying disease severity rather than direct contributors to pouch inflammation. It is possible that persistent immune dysregulation prior to surgery continues to influence postoperative outcomes (38, 39). In addition, prolonged exposure to immunosuppressive therapy may alter mucosal immune responses or microbial composition, both of which could potentially affect inflammatory processes within the ileal pouch.

The relationship between current smoking and pouch outcomes remains somewhat complex. Although smoking has historically been described as having a protective effect in UC, its role after IPAA is less clear (40). Smoking is known to influence mucosal immunity, epithelial barrier function, and intestinal microbial composition. These biological effects may modify inflammatory responses within the ileal pouch. The present findings suggest that smoking could be associated with an increased risk of chronic pouchitis, although the precise mechanisms remain uncertain and warrant further investigation (41).

The role of age in chronic pouchitis appears less definitive. While the pooled analysis initially indicated a relationship between increasing age and chronic pouchitis, this association was attenuated after accounting for potential publication bias (42). Differences in study design, patient populations, and statistical modeling of age across the included studies may partly explain this variability (43). Age was associated with chronic pouchitis in the primary analysis; however, this association became non-significant after trim-and-fill adjustment, suggesting that the finding should be interpreted with caution.

Female sex was also associated with an increased risk of chronic pouchitis in this analysis. The underlying reasons for this observation remain unclear. Sex-related differences in immune regulation, hormonal influences, and microbiota composition have been suggested as potential explanations for variations in inflammatory responses between males and females (44). Hormonal factors may influence mucosal immune activity and cytokine signaling, which could potentially affect susceptibility to chronic inflammation (45). Nevertheless, the current evidence remains limited, and further studies are needed to better understand the potential role of sex-related biological factors in the development of chronic pouchitis.

From a clinical perspective, the identification of factors potentially associated with chronic pouchitis may assist clinicians in recognizing patients who could benefit from closer postoperative follow-up. Individuals with primary sclerosing cholangitis, extraintestinal manifestations, or a history of intensive immunosuppressive therapy may represent a subgroup with a higher probability of developing chronic pouch inflammation. Early identification of these patients may facilitate more individualized postoperative management.

### Strengths and limitations

This study has several strengths. It provides a comprehensive synthesis of available evidence on risk factors for chronic pouchitis following IPAA and includes a relatively large, pooled sample size. Multiple potential predictors were evaluated, and sensitivity analyses were performed to assess the stability of the findings. Furthermore, publication bias was assessed using both funnel plots and Egger's test, and the trim-and-fill method was applied when bias was detected.

Nevertheless, several limitations should be acknowledged. Most of the included studies were observational, which may introduce selection bias and residual confounding. Variability in the definitions of chronic pouchitis and differences in study design may have contributed to heterogeneity. In addition, the number of studies available for certain risk factors was limited, reducing the statistical power of the analyses. Finally, evidence of publication bias was identified for age, indicating that this association should be interpreted cautiously. In addition, diagnostic criteria for chronic pouchitis varied across the included studies. Definitions included antibiotic-dependent pouchitis, antibiotic-refractory pouchitis, and recurrent pouchitis based on symptom frequency or endoscopic confirmation. This variability in diagnostic criteria may have introduced clinical heterogeneity and potentially affected the comparability of effect estimates across studies.

## Conclusion

Several clinical factors, including female sex, current smoking, primary sclerosing cholangitis, preoperative steroid or immunosuppressive use, and extraintestinal manifestations, are associated with an increased risk of chronic pouchitis after IPAA. The association between age and chronic pouchitis appears unstable and should be interpreted cautiously. These findings may assist in identifying high-risk patients and guiding postoperative surveillance; however, further prospective studies are needed to confirm these associations.

## Data Availability

The raw data supporting the conclusions of this article will be made available by the authors, without undue reservation.
