# Supplementary material for: Risk factors for chronic pouchitis in patients with ulcerative colitis following ileal pouch-anal anastomosis: a meta-analysis
Source: Front Med (Lausanne). 2026 Aug 17;13:1886122. doi: 10.3389/fmed.2026.1886122 (PMC13526607; doi:10.3389/fmed.2026.1886122)
Supplement: Supplementary file 1 [file Table_1.docx]

Table S1 search strategy

((("Colitis, Ulcerative"[Mesh]) OR (((((Colitis, Ulcerative[Title/Abstract]) OR (Colitis Gravis[Title/Abstract])) OR (Idiopathic Proctocolitis[Title/Abstract])) OR (Inflammatory Bowel Disease, Ulcerative Colitis Type[Title/Abstract])) OR (Ulcerative Colitis[Title/Abstract]))) AND (("Pouchitis"[Mesh]) OR (((Pouchitis[Title/Abstract]) OR (Pouch Ileitis[Title/Abstract])) OR (Ileitis, Pouch[Title/Abstract])))) AND (("Risk Factors"[Mesh]) OR (((((((((((((((((((Risk Factors[Title/Abstract]) OR (Factor, Risk[Title/Abstract])) OR (Risk Factor[Title/Abstract])) OR (Population at Risk[Title/Abstract])) OR (Populations at Risk[Title/Abstract])) OR (Risk Scores[Title/Abstract])) OR (Risk Score[Title/Abstract])) OR (Score, Risk[Title/Abstract])) OR (Risk Factor Scores[Title/Abstract])) OR (Risk Factor Score[Title/Abstract])) OR (Score, Risk Factor[Title/Abstract])) OR (Health Correlates[Title/Abstract])) OR (Correlates, Health[Title/Abstract])) OR (Social Risk Factors[Title/Abstract])) OR (Factor, Social Risk[Title/Abstract])) OR (Factors, Social Risk[Title/Abstract])) OR (Risk Factor, Social[Title/Abstract])) OR (Risk Factors, Social[Title/Abstract])) OR (Social Risk Factor[Title/Abstract])))

Table S2 GRADE Summary of Findings for Risk Factors of Chronic Pouchitis After IPAA

| Risk factor | Number of studies | Effect size (OR, 95% CI) | Certainty of evidence (GRADE) | Downgrade reasons | Interpretation |
| --- | --- | --- | --- | --- | --- |
| Female sex | 5 | 1.36 (1.05–1.76) | ⊕⊕◯◯ Low | Observational design, moderate heterogeneity, possible residual confounding | Probably associated with increased risk |
| Smoking | 6 | 2.46 (1.28–4.75) | ⊕⊕◯◯ Low | Observational bias, exposure measurement variability | Probably increases risk |
| Primary sclerosing cholangitis (PSC) | 5 | 2.30 (1.43–3.72) | ⊕⊕⊕◯ Moderate | Some inconsistency, but stable effect and low heterogeneity | Likely increases risk |
| Preoperative systemic steroid use | 6 | 1.61 (1.10–2.36) | ⊕⊕◯◯ Low | Confounding by disease severity, observational design | Probably associated with increased risk |
| Preoperative immunosuppressive therapy | 5 | 1.32 (1.02–1.70) | ⊕⊕◯◯ Low | Confounding, small effect size, heterogeneity | Uncertain to probably associated |
| Extraintestinal manifestations | 5 | 3.09 (1.65–5.77) | ⊕⊕⊕◯ Moderate | Some heterogeneity, but large effect size | Likely increases risk |
| Increasing age | 6 | 1.23 (1.03–1.47) → NS after trim-and-fill | ⊕◯◯◯ Very low | Publication bias, instability after trim-and-fill | Not reliable association |

Table S3. Study-specific definitions, follow-up, exposure coding, and covariate adjustment in the included studies

| Study | Definition and outcome ascertainment | Statistical model/modeled outcome | Variables included in the adjusted model | Indication for colectomy/IPAA | EIMs included or evaluated |
| --- | --- | --- | --- | --- | --- |
| Abdelrazeq et al., 2008 [16] | Diagnosis required combined clinical, endoscopic, and histologic criteria and the PDAI. Chronic pouchitis (CP) was defined as symptoms persisting continuously for >4 weeks despite full-dose standard therapy, or a need for >2 weeks of therapy each month for 3 consecutive months to maintain symptom control. Secondary causes were excluded. | Multivariable binary logistic regression specifically for CP; variables with univariable P < 0.10 were entered. | Age at initial surgery; extent of colitis; backwash ileitis; primary sclerosing cholangitis (PSC). | Medically refractory disease; fulminant colitis; dysplasia/colorectal cancer. | PSC was specifically evaluated and included in the adjusted model; other individual EIMs were not itemized in the model. |
| Akiyama et al., 2025 [17] | Clinical symptoms—increased stool frequency, rectal bleeding, fecal urgency/abdominal cramps, or fever—persisting for >4 weeks despite antibiotics and requiring long-term antibiotic and anti-inflammatory therapy. Relapse several months after temporary antibiotic withdrawal was also included. | Cox proportional-hazards and logistic regression models specifically for CP. | Cox model: age at colectomy; anastomosis type; focal pouch-body inflammation at the initial postoperative pouchoscopy; preoperative azathioprine/6-mercaptopurine; dysplasia/colorectal cancer; disease duration before surgery. Logistic model: age at colectomy; preoperative systemic steroids; medically refractory disease; abdominal abscess requiring drainage; overall afferent-limb involvement; inlet involvement; diffuse pouch-body inflammation; cuffitis; pouch-related fistula. | Medically refractory disease vs dysplasia/colorectal cancer. Both variables were considered separately in the CP models. | No composite or individual EIM variable was reported in the adjusted models. |
| Baek et al., 2025 [18] | CP was defined as persistent symptoms despite 4 weeks of conventional antibiotics. Chronic antibiotic-dependent pouchitis (CADP) was defined as symptomatic/endoscopic response but >4 recurrences per year requiring persistent antibiotics. Chronic antibiotic-refractory pouchitis (CARP) was defined as no response to conventional antibiotics and a need for >4 weeks of 5-aminosalicylates, steroids, immunomodulators, or biologic/small-molecule therapy. | Multivariable Cox model for CARP only; the composite CP comparison was unadjusted. | Age at UC diagnosis; smoking status (current and former vs nonsmoker); previous biologic/small-molecule use; previous immunomodulator use. | Steroid-refractory/acute fulminant disease; steroid-dependent disease; dysplasia/colorectal cancer; obstruction; perforation; toxic megacolon; massive hemorrhage. | PSC; arthralgia; pyoderma gangrenosum; erythema nodosum; and other reported EIMs. These were described at baseline but were not retained in the reported adjusted CARP model. |
| Barnes et al., 2021 [19] | Claims-based pouchitis required preoperative UC and IPAA codes plus either a pouchitis diagnosis code or a ciprofloxacin/metronidazole prescription within 2 years. Isolated acute pouchitis was one episode; recurrent pouchitis was at least one recurrence during follow-up. The claims algorithm was not designed to identify CP; recurrent pouchitis was therefore the nearest chronicity measure. | Multivariable logistic regression for any pouchitis, not specifically recurrent pouchitis or CP. | Indication for colectomy; sex; age group; PSC; smoking/tobacco use; Deyo–Charlson comorbidity score; US region; insurance plan type; index year; pre-colectomy mesalamine, immunomodulator, anti-tumor necrosis factor, and corticosteroid use. | Medically refractory disease; emergency colectomy; dysplasia/cancer. Indication was included in the adjusted model. | PSC was evaluated as a specific comorbidity; a broader composite EIM variable was not used. |
| Bertucci Zoccali et al., 2019 [20] | Pouchitis was diagnosed using PDAI clinical criteria—frequency, urgency, rectal bleeding, and fever—and/or typical endoscopic findings. No duration- or treatment-response-based definition of CP was specified. | Binary logistic regression for any pouchitis, not CP; variables with univariable P < 0.15 were entered. | Preoperative oral steroids; 5-aminosalicylates; immunomodulators; anti-tumor necrosis factor therapy; number of surgical stages. | Indication was recorded, but refractory disease and neoplasia-related indications were not separately included in the final model. | EIMs were not included in the reported adjusted model, and individual EIM components were not specified. |
| Ferrante et al., 2008 [21] | Diagnosis required typical symptoms plus suggestive pouch endoscopy and confirmatory histology. Acute pouchitis responded rapidly and lasted <4 weeks; acute-relapsing pouchitis comprised ≥3 acute episodes; CP comprised symptoms lasting >4 weeks despite standard therapy. | Cox proportional-hazards model specifically for CP; variables with Breslow P< 0.10 were entered. | Extraintestinal manifestations (EIMs); backwash ileitis; anti-chitobioside carbohydrate antibodies; anti-outer-membrane porin antibodies; TLR2 R753Q genotype; preoperative aminosalicylate use; age at proctocolectomy. | Predominantly medically refractory UC; dysplasia/colorectal cancer was also represented. Indication was not retained in the reported CP model. | A composite preoperative EIM variable was entered into the CP model. Individual EIM components were not itemized in the adjusted-model report. |
| Fleshner et al., 2007 [22] | Pouchitis was a clinical syndrome confirmed endoscopically. Acute flares responded to antibiotics, were ≥4 months apart, and were separated by return to the usual bowel pattern. CP required continuous antibiotics for symptom relief or was nonresponsive to antibiotics; pouchoscopy/biopsy and evaluation for alternative causes were performed. | Stepwise multivariable logistic regression specifically for CP. | Candidate variables: female sex; preoperative thrombocytosis; nonsmoking; backwash ileitis; EIMs; follow-up duration. The final model retained EIMs, thrombocytosis, follow-up duration, and smoking history (current/former vs never). | Medically refractory disease and neoplasia-related indications were represented, but indication was not included in the final CP model. | Composite preoperative EIM status was used. Individual rheumatologic, cutaneous, ocular, or hepatobiliary manifestations were not separately modeled. |
| Gorrepati et al., 2018 [23] | Pouchitis was identified by relevant symptoms and/or typical endoscopic/histologic inflammation. CP required persistence of symptoms and/or endoscopic/histologic findings for ≥4 weeks. Antibiotic dependence meant >1 consecutive antibiotic course; antibiotic-refractory disease never responded to antibiotics. | Multivariable logistic regression for any pouchitis, not the CP subgroup. | Current and former tobacco use; female sex; three-stage surgery; preoperative antibiotic, immunomodulator, biologic, and corticosteroid use; PSC; pyoderma gangrenosum; anxiety/depression. | Indication for colectomy was not reported as a covariate in the adjusted model. | PSC and pyoderma gangrenosum were evaluated as separate covariates. |
| Hashavia et al., 2012 [24] | CP was defined as ≥4 weeks of persistent symptoms and included antibiotic-dependent disease—prolonged therapy with >2 antibiotics—and antibiotic-refractory disease. All CP cases had pouch endoscopy and biopsy confirmation of acute inflammation; pouchitis was defined as PDAI >7. | Forced-entry multiple logistic regression specifically for CP. | Sex; ethnicity; age at operation; number of operative stages; diverting ileostomy; age at disease onset; disease duration; family history of inflammatory bowel disease; extent of colitis; indication for restorative proctocolectomy; preoperative treatment group; EIMs; PSC; follow-up duration. | Indication for restorative proctocolectomy was included as a candidate covariate; the detailed categories were not reported in the adjusted-model table. | Composite EIM status and PSC were evaluated separately; other individual EIM components were not itemized. |
| Hata et al., 2017 [25] | Pouchitis was defined as modified PDAI ≥5. CP included relapse after antibiotic withdrawal requiring >4 weeks of antibiotics, or nonresponse to a 4-week antibiotic course requiring stronger therapy—5-aminosalicylates, topical steroids, immunomodulators, cytapheresis, or anti-tumor necrosis factor therapy. Crohn’s disease of the pouch was excluded. | Cox proportional-hazards model specifically for CP; variables with univariable P < 0.20 were entered. | Preoperative EIMs; extent of UC (total vs left-sided colitis). | Refractory disease, fulminant disease, and cancer/dysplasia-related surgery were represented; indication was not retained in the CP model. | Preoperative EIMs included rheumatologic, cutaneous, ocular, and hepatobiliary manifestations, including peripheral arthritis/sacroiliitis or ankylosing spondylitis, pyoderma gangrenosum, uveitis, and PSC. The model used composite EIM status rather than individual disorders. |
| Hata et al., 2003 [26] | Pouchitis was defined as PDAI ≥7; ulcers confined to the staple line or rectal remnant were excluded. Recurrent pouchitis was reported in four patients, but no operational definition of CP was provided. | Multivariable Cox model for time to first/any pouchitis, not CP. | Sex; age at UC onset; smoking history within 7 years; EIMs; operation type (handsewn with mucosectomy vs double-stapled without mucosectomy). | Indication for colectomy was not included in the reported adjusted model. | Composite EIM status was evaluated; individual manifestations were not separately reported in the model. |
| Kasashima et al., 2025 [27] | Idiopathic pouchitis required clinical symptoms—such as increased stool frequency, urgency, or pelvic discomfort—and endoscopic findings after exclusion of secondary causes. CP was defined as persistent or recurrent symptoms requiring prolonged antibiotic therapy or immunosuppressive treatment. | Multivariable regression and predictive modeling for hospitalization-requiring/any pouchitis, not specifically CP. | Age; body mass index; corticosteroid use within 1 month before surgery; immunosuppressive therapy within 1 month before surgery. | Refractory disease; cancer/high-grade dysplasia; severe/fulminant colitis; massive bleeding; perforation; toxic megacolon; EIM-related indications, including PSC; and other indications. | EIMs were not evaluated as a composite predictor. An extraintestinal complication, including PSC, could constitute an indication for surgery, but individual EIMs were not analyzed separately as pouchitis predictors. |
| Okita et al., 2013 [28] | Pouchitis was defined as modified PDAI ≥5 after exclusion of secondary causes. CP combined antibiotic-dependent disease requiring long-term continuous antibiotics, antibiotic-refractory disease, and relapsing disease (>3 episodes/year or recurrence within 1 month of antibiotic withdrawal). Acute pouchitis lasted <4 weeks and improved with antibiotics. | Multivariable Cox proportional-hazards model specifically for CP; variables with univariable P < 0.20 were entered. | Disease duration <12 years; cumulative pre-colectomy steroid dose ≥7.5 g; steroid dose ≥500 mg/month immediately before colectomy. | Indication for surgery was assessed, but the exact refractory disease vs dysplasia/cancer categories were not reported in the final adjusted model. | Composite EIM status was assessed but was not retained in the final model; individual EIM components were not itemized. |

Figure S1 Sensitivity analysis results of increasing age

Figure S2 Sensitivity analysis results of smoking

Figure S3 Sensitivity analysis results of extraintestinal manifestations

Figure S4 Funnel plot of meta-analysis of increasing age

Figure S5 Funnel plot of meta-analysis of female

Figure S6 Funnel plot of meta-analysis of smoking

Figure S7 Funnel plot of meta-analysis of primary sclerosing cholangitis

Figure S8 Funnel plot of meta-analysis of preoperative systemic steroids

Figure S9 Funnel plot of meta-analysis of preoperative immune drugs

Figure S10 Funnel plot of meta-analysis of extraintestinal manifestations

Figure S11 Trim-and-fill analysis results of increasing age
